# The Impact of Age on the Post-operative Outcomes in Patients Undergoing Resection for Oesophageal and Gastric Cancer

**DOI:** 10.1007/s00268-023-07223-x

**Published:** 2023-10-18

**Authors:** Cameron Law, Nazim Bhimani, David Mitchell, Mia Yue Yu, Priscilla Chan, Steven Leibman, Garett Smith

**Affiliations:** 1 Upper Gastrointestinal Surgical Unit, Clinical Administration 8A Office ASB, Royal North Shore Hospital, Reserve Road, St Leonards, NSW 2065 Australia; 2 Northern Clinical School, University of Sydney, Sydney, NSW Australia; 3 Faculty of Medicine and Health, University of Sydney, Sydney, NSW Australia

## Abstract

**Background:**

Within our ageing population, there is an increasing number of elderly patients presenting with oesophagogastric cancer. Resection remains the mainstay of curative treatment however it has substantial morbidity. The aim of this study was to assess whether age was an independent predictor of resection related complications in our unit.

**Methods:**

A retrospective cohort study of prospectively collated data from 2002 to 2020 of patients undergoing resection for oesophageal and gastric cancers was analysed. Patients aged over 75 and 75 and under were compared for peri-operative morbidity (via the Clavien-Dindo classification), length of stay (LOS), unplanned readmission, 30- and 90-day mortality, and use of neoadjuvant therapy.

**Results:**

Data for 466 consecutive patients undergoing oesophagogastric resection (277 oesophagectomy and 189 gastrectomy) were available for analysis. 22% of patients were aged over 75 (14% (39/277) of the oesophagectomy cohort, 34% (65/189) of the gastrectomy cohort). Oesophagectomy patients over 75 were more likely to develop post-operative complications, particularly cardiac or thromboembolic, (69.2%) than those in the younger cohort (50.4%, *p* = 0.029). There was no difference in complication rates between the younger and older patients undergoing gastrectomy (29.0% vs. 33.9% *p* = 0.495). The 30- and 90-day mortality rates were 1.4% (*n* = 4) and 2.5% (*n* = 7), respectively, for the oesophagectomy cohort and 1.1% (*n* = 2) and 1.6% (*n* = 3) for the gastrectomy cohort, with no difference between age groups.

**Conclusion:**

In this series, we found that patients over the age of 75 were able to undergo oesophageal and gastric resection with curative intent with acceptable post-operative morbidity and mortality.

## Introduction

As the life expectancy of our population increases, there is a greater incidence of oesophagogastric cancer, particularly in the elderly population [1, 2]. Treatment is complex and resource intensive and usually includes adjuvant therapy combined with complex foregut resection [3–5].

Foregut resection in the elderly is generally undertaken with caution due to increased frailty, greater comorbidities and impaired nutritional status. While it would be reasonable to assume that older patients undergoing oesophagogastric resection would have higher rates of perioperative complication [6, 7], the available evidence is limited and conflicting. Some retrospective studies support the idea that elderly patients suffer more post-operative medical complications [8], and subsequently higher rates of mortality [9], though opposing evidence indicates comparable morbidity and mortality rates [10, 11]. Available data are further confounded by the use of adjuvant therapies which may result in higher complication rates in the elderly and therefore are frequently omitted [12–14].

The aim of this study was to ascertain whether increased age, over 75 years, has an adverse effect on post-operative, short-term, outcomes in patients undergoing oesophagogastric resection.

## Materials and methods

A single unit cohort study of patients undergoing curative resection for oesophagogastric cancer between 2002 and 2020 at two co-located hospitals was undertaken (Royal North Shore and North Shore Private hospitals). Ethics approval was given by the Human Research Ethics Committee of the New South Wales Northern Sydney Local Health District, HREC reference number 2022/ETH00802. Patients with oesophagogastric cancer who underwent curative surgical resection in our unit were retrospectively identified from a prospectively collated database. Patients undergoing resection for benign disease or where concurrent laryngeal resection was performed were excluded from the study.

The database included patient characteristics (age, sex, smoking status), the American Society of Anesthesiologists (ASA) grade of the patient [15], comorbidities, operative details, use of adjuvant therapy, type and stage of disease. Post-operative data included length of stay (LOS), unplanned return to theatre, unplanned ICU admission, unplanned reintubation, unplanned readmission to hospital, in-hospital mortality, and 30- and 90-day mortality. Specific surgical complications recorded included anastomotic leak, haemorrhage, intra-abdominal abscess, wound infection and chylothorax. Non-surgical complications included respiratory, cardiac and thrombo-embolic. Collected data were examined with a focus on age greater than 75 years, and 75 years or younger.

Treatment and perioperative data collected early in this series were based on the data set from the Association of Upper Gastrointestinal Surgeons of the United Kingdom and Ireland (AUGIS), and severity of complications was recorded using the Clavien-Dindo grading system [16]. Later in the series, details of complications were recorded according to the International Oesophagectomy Complications Consensus Group [17].

Pre-treatment staging comprised of CT and FDG PET scans. Staging laparoscopy was performed for those patients with gastric or junctional cancer. Patients were routinely discussed at a multidisciplinary team meeting. Patients with a gastric malignancy, or a Siewart type 3 oesophagogastric junctional cancer, underwent a total or subtotal gastrectomy. Oesophageal resection in this series was either via open Ivor Lewis resection or hybrid thoracoscopic 3 stage resection dependent on tumour location. Thoracic epidural and on table extubation were routine. Surgery was performed by the two senior authors (SL and GS) or trainees under their supervision.

Categorical variables were presented as counts and percentages and analysed using Fishers’ exact or Chi-square tests. Continuous variables were expressed as mean and standard deviation for normally distributed data and analysed using T tests. Continuous variables which were not normally distributed were expressed as median and range and analysed using Mann–Whitney U test. Association of variables with complications following oesophagectomy were assessed using logistic regression. All univariable analyses with *p* value < 0.2 were included in the multivariable model after testing for collinearity. Backward elimination was used as the variable selection method. The results were presented with an odds ratio (OR), 95% confidence interval (CI) and a corresponding *p* value.

## Results

There were 466 consecutive patients included in this analysis of which 277 underwent oesophagectomy and 189 underwent gastrectomy. Of these patients, 22% were aged over 75 (*n* = 104); 14% of the oesophageal cohort (*n* = 39/277) and 34% of the gastric cohort (*n* = 65/189). Patient characteristics are outlined in Tables 1 and 2. Patients aged over 75, in both the oesophagectomy and gastrectomy cohorts, were more likely to be ASA grade 3 or higher (*p* = 0.002 and *p* = 0.005, respectively) and more frequently had pre-existing cardiovascular and neurological comorbidities. Tables 3 and 4 outline the treatment received, the histopathology and staging (TNM) of both oesophagectomy and gastrectomy patients. There were 103 patients with oesophageal cancer who underwent an Ivor Lewis resection, and 174 patients had a hybrid 3-stage resection (Table 3 37% vs. 63%). Amongst those with gastric cancer, 77 patients underwent a total gastrectomy, and 112 had either a subtotal (*n* = 104) or a distal gastrectomy (*n* = 8) (Table 4 41% vs. 59%).

**Table 1:** Oesophagectomy patient characteristics by age

	Total population (*n* = 277)	Age ≤ 75 (*n* = 238)	Age > 75 (*n* = 39)	*p* value
Median Age (Range)	67(34–85)			
Sex, *n* (%)				0.960
Male	221(79.8)	190(79.8)	31(79.5)	
Female	56(20.2)	48(20.2)	8(20.5)	
ASA Grade, *n* (%)				0.002
1 or 2	157(56.7)	144(60.5)	13(33.3)	
3 or 4	120(43.3)	94(39.5)	26(66.7)	
COPD, *n* (%)	33(11.9)	25(10.5)	8(20.5)	0.074
HTN, *n* (%)	78(28.2)	68(28.6)	10(25.6)	0.706
CAD, *n* (%)	41(14.8)	30(12.6)	11(28.2)	0.011
Arrhythmia, *n* (%)	28(10.1)	20(8.4)	8(20.5)	0.020
Diabetes, *n* (%)	33(11.9)	29(12.2)	4(10.3)	0.999
PE/DVT, *n* (%)	12(4.3)	10(4.2)	2(5.1)	0.679
TIA/CVA, *n* (%)	10(3.6)	5(2.1)	5(12.8)	0.001
Smoking, *n* (%)	20(7.2)	20(8.4)	0(0.0)	0.088
Renal, *n* (%)	6(2.2)	6(2.5)	0(0.0)	0.600

*ASA* American Society of Anesthesiologists; *COPD* Chronic Obstruction Pulmonary Disease; *HTN* Hypertension; *CAD* Coronary artery disease; *PE* Pulmonary embolism; *DVT* Deep venous thrombosis; *TIA* Transient ischaemic attack; *CVA* Cerebrovascular accident

**Table 2:** Gastrectomy patient characteristics by age

	Total population (*n* = 189)	Age ≤ 75 (*n* = 124)	Age > 75 (*n* = 65)	*p* value
Median Age (Range)	71(30–91)			
Sex, *n* (%)				0.088
Male	112(59.3)	68(54.8)	44(67.7)	
Female	77(40.7)	56(45.2)	21(32.3)	
ASA Grade, *n* (%)				0.005
1 or 2	108(57.1)	80(64.5)	28(43.1)	
3 or 4	81(42.9)	44(35.5)	37(56.9)	
COPD, *n* (%)	4(2.19)	2(1.6)	2(3.1)	0.609
HTN, *n* (%)	59(31.2)	31(25.0)	28(43.1)	0.011
CAD, *n* (%)	22(11.6)	10(8.1)	12(18.5)	0.034
Arrhythmia, *n* (%)	15(7.9)	6(4.8)	9(13.9)	0.030
Diabetes, *n* (%)	24(12.7)	13(10.5)	11(16.9)	0.207
PE/DVT, *n* (%)	9(4.8)	4(3.2)	5(7.7)	0.279
TIA/CVA, *n* (%)	6(3.2)	1(0.8)	5(7.7)	0.019
Smoking, *n* (%)	15(7.9)	12(9.7)	3(4.6)	0.269
Renal, *n* (%)	7(3.7)	3(2.4)	4(6.2)	0.235

*ASA* American Society of Anaesthesiologists; *COPD* Chronic Obstruction Pulmonary Disease; *HTN* Hypertension; *CAD* Coronary artery disease; *PE* Pulmonary embolism; *DVT* Deep venous thrombosis; *TIA* Transient ischaemic attack; *CVA* Cerebrovascular accident

**Table 3:** Oesophagectomy treatment and histopathological details by age

	Total population (*n* = 277)	Age ≤ 75 (*n* = 238)	Age > 75 (*n* = 39)	*p* value
Neoadjuvant therapy, *n* (%)				0.753
Chemo-radiotherapy	120(43.3)	103(43.3)	17(43.6)	
Chemotherapy	97(35.0)	85(35.7)	12(30.8)	
None	60(21.7)	50(21.0)	10(25.6)	
Adjuvant chemotherapy, *n* (%)	43(15.5)	39(16.4)	4(10.3)	0.327
Hospital, *n* (%)				0.296
Public	76(27.4)	68(28.6)	8(20.5)	
Private	201(72.6)	170(71.4)	31(79.5)	
Procedure, *n* (%)				0.860
Ivor Lewis	103(37.2)	88(37.0)	15(38.5)	
3 Stage	174(62.8)	150(63.0)	24(61.5)	
Pathological T Stage, *n* (%)				0.34
T0	28(10.1)	24(10.1)	4(10.3)	
T1	80(28.9	64(26.9	16(41.0	
T2	42(15.1)	36(15.1)	6(15.4)	
T3	114(41.2)	103(43.3)	11(28.2)	
T4	13(4.7)	11(4.6)	2(5.1)	0
Pathological N Stage, *n* (%)				0.96
N0	157(56.7)	134(56.3)	23(59.0)	
N1	79(28.5)	69(29.0)	10(25.6)	
N2	25(9.0)	21(8.8)	4(10.3)	
N3	16(5.8)	14(5.9)	2(5.1)	0
Histopathology, *n* (%)				0.325
Adenocarcinoma	227(82.0)	192(80.7)	35(89.7)	
Squamous cell carcinoma	43(15.5)	40(16.8)	3(7.7)	
Other	7(2.5)	6(2.5)	1(2.6)

**Table 4:** Gastrectomy treatment and histopathological details by age

	Total population (*n* = 189)	Age ≤ 75 (*n* = 124)	Age > 75 (*n* = 65)	*p* value
Neoadjuvant chemotherapy, *n* (%)	71(37.6)	55(44.4)	16(24.6)	0.008
Adjuvant chemotherapy, *n* (%)	62(32.8)	55(44.4)	7(10.8)	< 0.001
Hospital, *n* (%)				0.658
Public	76(40.6)	51(41.8)	25(38.5)	
Private	111(59.4)	71(58.2)	40(61.5)	
Gastrectomy, *n* (%)				0.163
Distal or Subtotal	112(59.3)	69(55.7)	43(66.2)	
Total or Extended total	77(40.7)	55(44.3)	22(33.8)	
Lymph node dissection, *n* (%)				0.145
D1 or D1 +	139(73.5)	87(70.2)	52(80.0)	
D2	50(26.5)	37(29.8)	13(20.0)	
Pathological T Stage, *n* (%)				0.191
T0	9(4.8)	7(5.6)	2(3.1)	
T1	55(29.1)	41(33.1)	14(21.5)	
T2	32(16.9)	16(12.9)	16(24.6)	
T3	62(32.8)	41(33.1)	21(32.3)	
T4	31(16.4)	19(15.3)	12(18.5)	
Pathological N Stage, *n* (%)				0.196
N0	96(50.8)	63(50.8)	33(50.8)	
N1	37(19.6)	22(17.8)	15(23.1)	
N2	24(12.7)	20(16.1)	4(6.1)	
N	32(16.9)	19(15.3)	13(20.0)	
Histopathology, *n* (%)				0.301
Adenocarcinoma	179(94.7)	115(92.8)	64(98.5)	
Neuroendocrine	3(1.6)	3(2.4)	0(0.0)	
Other	7(3.7)	6(4.8)	1(1.5)	

### Oesophagectomy cohort

Older patients undergoing oesophagectomy were as likely to receive adjuvant therapy as the younger cohort (Table 3). LOS in the older group was 17 days versus 15 days in the younger cohort although there was no statistical difference (*p* = 0.301).

There was no difference in mortality rates between the two cohorts; 30- and 90-day mortality rates in the older cohort were 2.6% and 2.6% (*n* = 1) and in the younger cohort 1.3% (*n* = 3) and 2.5% (*n* = 6), respectively (Table 5).

**Table 5:** Oesophagectomy outcomes by age

	Total population (*n* = 277)	Age ≤ 75 (*n* = 238)	Age > 75 (*n* = 39)	*p* value
Overall post-op Complications, *n* (%)	147 (53.1)	120 (50.4)	27 (69.2)	0.029
Intra-op Complications, *n* (%)	6 (2.2)	5 (2.1)	1 (2.6)	0.999
Surgical Complications, *n* (%)	96 (34.7)	83 (34.9)	13 (33.3)	0.851
Non-Surgical Complications, *n* (%)	97 (35.0)	74 (31.1)	23 (59.0)	0.001
Blood Transfusion, *n* (%)	47 (17.0)	37 (15.6)	10 (25.6)	0.120
Unplanned return to OR, *n* (%)	88 (31.8)	77 (32.3)	11 (28.2)	0.606
Unplanned ICU admission, *n* (%)	44 (15.9)	35 (14.7)	9 (23.1)	0.185
Unplanned readmission, *n* (%)	17 (6.1)	16 (6.7)	1 (2.6)	0.482
Reintubation required, *n* (%)	21 (7.6)	18 (7.6)	3 (7.7)	0.999
Tracheostomy required, *n* (%)	4 (1.4)	4 (1.7)	0 (0.0)	0.999
In-hospital Mortality, *n* (%)	6 (2.2)	5 (2.1)	1 (2.6)	0.999
30-day Mortality, *n* (%)	4 (1.4)	3 (1.3)	1 (2.6)	0.457
90-day Mortality, *n* (%)	7 (2.5)	6 (2.5)	1 (2.6)	0.999
Median LOS (days) and range	15 (5–160)	15 (5–160)	17 (8–67)	0.301
Clavien Dindo Grade, *n* (%)				0.795
Grade 1	9 (6.1)	7 (5.8)	2 (7.4)	
Grade 2	46 (31.3)	36 (30.0)	10 (37.0)	
Grade 3a or 3b	68 (46.3)	58 (48.4)	10 (37.0)	
Grade 4a or 4b	19 (12.9)	15 (12.5)	4 (14.8)	
Grade 5	5 (3.4)	4 (3.3)	1 (3.7)	

*LOS* Length of stay

Patients over 75 were more likely to develop post-operative complications (*p* = 0.029), and they were more likely to be non-surgical (*p* = 0.001) (Table 5). Of those non-surgical complications cardiac (specifically cardiac arrythmias) and thrombo-embolic (DVT/PE) adverse events were comparatively more common (*p* =  < 0.001 and *p* = 0.017 respectively) (Appendix Table 8).

There was no difference in rates of surgical complications between age groups of oesophagectomy patients (Appendix Table 9). A conduit or anastomotic leak occurred in 39/277 patients (14.1%), 8/103 (7.8%) of whom had an Ivor Lewis versus 31/174 (17.2%) who had a 3-stage oesophagectomy (*p* = 0.020). Leaks in the 3-stage oesophagectomy group were predominately due to necrosis of the tip of the conduit rather than a leak from the anastomosis.

Univariable analyses are presented in Appendix Tables 10 and 11 for all complications and non-surgical complications for oesophagectomy patients. On multivariable analysis, ASA grade 3 or 4 (OR 1.85, 95% CI 1.13–3.03, *p* = 0.015), an operation performed in our public hospital (OR 1.78, 95% CI 1.02–3.11, *p* = 0.043) and a 3-stage oesophagectomy (OR 1.97, 95% CI 1.19–3.26, p = 0.009) were predictive for increased rates of all post-operative complications (surgical and non-surgical). Whilst age over 75 years, surgery performed in our public hospital and a 3-stage oesophagectomy, were predictive of non-surgical complications (Table 6 and Appendix Table 12).

**Table 6:** Oesophagectomy; multivariable analysis for all complications

	Odds Ratio	95% CI	*p* value
ASA Grade			0.015
1 or 2	Reference		
3 or 4	1.85	1.13–3.03	
Hospital			0.043
Private	Reference		
Public	1.78	1.02–3.11	
Procedure			0.009
Ivor Lewis	Reference		
3 Stage	1.97	1.19–3.26	

*ASA* American Society of Anaesthesiologists

### Gastrectomy cohort

Gastrectomy patients more likely received neoadjuvant or adjuvant systemic therapy if they were 75 years or younger (*p* = 0.008 and *p* =  < 0.001) (Table 4). Patients over 75 had a greater LOS (median 13 days vs. 12 days, *p* = 0.006). There was no difference in post-operative complications between age groups (Table 7). An anastomotic leak occurred in 16/189 patients (8.5%); 10/124 (8.1%) of those patients were aged 75 years or under, and 6/65 (9.2%) were over 75 (*p* = 0.784).

**Table 7:** Gastrectomy outcomes by age

	Total population (*n* = 189)	Age ≤ 75 (*n* = 124)	Age > 75 (*n* = 65)	*p* value
Overall post-op Complications, *n* (%)	58(30.7)	36(29.0)	22(33.9)	0.495
Intra-op Complications, *n* (%)	4(2.1)	1(0.8)	3(4.6)	0.118
Surgical Complications, *n* (%)	32(16.9)	22(17.7)	10(15.4)	0.681
Non-Surgical Complications, *n* (%)	44(23.3)	25(20.2)	19(29.2)	0.161
Unplanned return to OR, *n* (%)	15(7.9)	9(7.3)	6(9.2)	0.634
Unplanned ICU admission, *n* (%)	21(11.1)	13(10.5)	8(12.3)	0.705
Unplanned admission, *n* (%)	13(6.9)	10(8.1)	3(4.6)	0.548
In-hospital Mortality, *n* (%)	2(1.1)	1(0.8)	1(1.5)	0.999
30-day Mortality, *n* (%)	2(1.1)	1(0.8)	1(1.5)	0.999
90-day Mortality, *n* (%)	3(1.6)	1(0.8)	2(3.1)	0.272
Median LOS (days) and range	12(7–232)	12(7–232)	13(8–172)	0.006
Clavien Dindo Grade, *n* (%)				0.650
Grade 1	8(13.8)	6(16.7)	2(9.1)	
Grade 2	28(48.3)	15(41.7)	13(59.1)	
Grade 3a or 3b	11(19.0)	7(19.4)	4(18.2)	
Grade 4a or 4b	9(15.5)	7(19.4)	2(9.1)	
Grade 5	2(3.4)	1(2.8)	1(4.5)	

*LOS* Length of stay

There was no difference between age groups in rates or severity of complications, and 30- and 90- day mortality. 30- and 90-day mortality rates in the older cohort were 1.5% (*n* = 1) and 3.1% (*n* = 2) and in the younger cohort 0.8% and 0.8% (*n* = 1) respectively (Table 7).

## Discussion

The risk of complications following treatment for oesophgogastric malignancy is not insignificant and overall survival rates are low [18, 19]. The influence of the patient’s age as a predictor of complication following foregut resection would seem likely from an intuitive viewpoint. The literature regarding this however has been contradictory; some suggest advanced age is a predictor for morbidity and mortality [9, 20] while others do not corroborate this [10, 11, 21]. Surgeons should be pragmatic in their approach to the elderly patient when offering surgical resection, with particular consideration of patients’ functional baseline [22] as well as their pre-existing comorbidities. Providing accurate peri-operative mortality and complication rates to patients, allowing informed decision making, is essential. The aim of our study was therefore to scrutinise short-term outcomes in elderly patients undergoing oesophagogastric resection within our institution.

In our series, elderly patients were more likely to have a higher ASA grade and more pre-treatment comorbidities. Advanced age was a predictor for non-surgical complications in those undergoing an oesophagectomy, specifically they were more likely to develop cardiac arrythmia and thromboembolic complications. Prophylactic beta blockade has been purported to prevent atrial fibrillation following oesophagectomy, however, due to conflicting evidence for and against the use of beta blockers, our institution has not adopted this for routine practice [23–25]. Despite some evidence for prolonged use of post-discharge DVT prophylaxis it has not been the policy of our treating group to mandate post-discharge DVT prophylaxis in all our resected patients. It is well recognised that patients undergoing neoadjuvant chemotherapy develop deep venous thrombosis during their treatment [26] and our results raise the question as to whether routine screening for DVT may be appropriate following neoadjuvant chemotherapy. Our data show that increased age did not confer an increased risk of complications in patients undergoing gastrectomy. At our institution, for patients undergoing oesophagogastric resection, it appears that age has not been a factor in determining post-operative mortality.

Along with higher ASA grade and type of oesophageal resection, patients undergoing oesophageal resection in our public facility had higher complication rates than patients in our private facility. Trainee surgeons performing oesophagogastric resection were supervised by the consultant surgeon authors. The surgical and anaesthetic approach and post-operative care were identical in both co-located facilities. No other factors predicting a higher risk of complication were able to be identified in this group of patients. This disparity in complication rates is the subject of ongoing evaluation in our unit.

Curative treatment of oesophagogastric malignancy is challenging for patients, carers, and health care professionals. Overall 5-year survival rates for oesophageal cancer are around 10–22% [27], and 33% for gastric cancer [28]. Identifying patients in whom rates of complication may be sufficiently high to not recommend curative treatment is critical.

The authors recognise limitations to this study, including the retrospective design. This was mitigated by prospective collection of data and therefore no recall bias, no exclusion of appropriate patients and a large study size. The study spanned 18 years with standardised surgical data collected by two surgeons throughout. During this timeframe however the treatment paradigm for these malignancies has evolved and we understand this may have impacted the peri-operative outcomes. The data are from a single institution, and future work could look to collaborate in a multi-centre approach. It has not been our practice to routinely assess patient frailty prior to offering treatment however, there is an increasing body of evidence that frailty may be a more accurate predictor of perioperative outcome than age [29–31]. A number of clinical and radiological scoring systems have been developed with a view to risk stratification [32–34], an example being the Edmonton Frail Scale which is a validated and practical screening tool for frailty [35]. Perhaps a specific adaptation of these methods that is applicable to geriatric patients undergoing workup for curative treatment of oesophagogastric malignancy may become clinically relevant in the future. An obvious limitation of our study is that of selection bias. All patients referred to our unit for consideration of curative resection were presented at our Multidisciplinary team meeting. Not all patients with localised disease within our referral network were discussed at our meeting. Conceivably, if an elderly patient was diagnosed with oesophageal malignancy and it was felt by the diagnosing gastroenterologist or the patient’s general practitioner that the patient was unfit for surgery, they may not have been referred for discussion. Unfortunately, it is therefore impossible to calculate a denominator of patients which includes patients over 75 with potentially curable disease who were not offered resection. While this absence of a denominator detracts from the strength of our study, we believe our conclusion that resection should be offered to selected elderly patients is valid.

## Conclusion

This study shows that curative surgical resection can be offered safely to elderly patients without significant adverse outcomes, or increased peri-operative morbidity, and with acceptable post-operative mortality rates. Age alone is not a reliable predictor of poor outcomes, and therefore our unit will continue to offer surgery, including the use of adjuvant therapies, to select patients over the age of 75. Improving methods for identifying patients at greater risk of complications following oesophagogastric resection should be a priority for clinicians into the future.

## Appendix

See Tables

**Table 8:** Oesophagectomy breakdown of non-surgical complications by age

	Total population (*n* = 277)	Age ≤ 75 (*n* = 238)	Age > 75 (*n* = 39)	*p* value
Cardiac Ischaemic event, *n* (%)	1 (0.4)	1 (0.4)	0 (0.0)	0.999
Cardiac Arrhythmia, *n* (%)	41 (14.8)	27 (11.3)	14 (35.9)	< 0.001
Other cardiovascular, *n* (%)	3 (1.1)	2 (0.8)	1 (2.6)	0.367
Pneumonia, *n* (%)	29 (10.5)	23 (9.7)	6 (15.4)	0.267
PE/DVT, *n* (%)	14 (5.1)	9 (3.8)	5 (12.8)	0.017
Other Pulmonary, *n* (%)	30 (10.8)	25 (10.5)	5 (12.8)	0.666
Renal, *n* (%)	2 (0.7)	2 (0.8)	0 (0.0)	0.999
CNS*, *n* (%)	7 (2.5)	4 (1.7)	3 (7.7)	0.060
Other non-surgical complication, *n* (%)	20 (7.2)	16 (6.7)	4 (10.3)	0.500

^*^6 patients with delirium, 1 with a seizure

*PE* Pulmonary embolism; *DVT* Deep venous thrombosis; *CNS* Central nervous system

8,

**Table 9:** Oesophageal surgical complications by age

	Total population (*n* = 277)	Age ≤ 75 (*n* = 238)	Age > 75 (*n* = 39)	*p* value
Anastomotic Leak, *n* (%)				0.621
Yes	39 (14.1)	35 (14.7)	4 (10.3)	
No	238 (85.9)	203 (85.3)	35 (89.7)	
Wound Infection, *n* (%)				0.758
Yes	19 (6.9)	16 (6.7)	3 (7.7)	
No	258 (93.1)	222 (93.3)	36 (92.3)	
Peritonitis, *n* (%)				0.999
Yes	1 (0.4)	1 (0.4)	0 (0.0)	
No	276 (99.6)	237 (99.6)	39 (100.0)	
Chylothorax, *n* (%)				0.999
Yes	16 (5.8)	14 (5.9)	2 (5.1)	
No	261 (94.2)	224 (94.1)	37 (94.9)	
Pleural Effusion, *n* (%)				0.223
Yes	25 (9.0)	24 (10.1)	1 (2.6)	
No	252 (91.0)	214 (89.9)	38 (97.4)	
Abscess, *n* (%)				0.999
Yes	2 (0.7)	2 (0.8)	0 (0.0)	
No	275 (99.3)	236 (99.2)	39 (100.0)	
Bleeding, *n* (%)				0.999
Yes	7 (2.5)	6 (2.5)	1 (2.6)	
No	270 (97.5)	232 (97.5)	38 (97.4)	
Jejunal Tube Complication, *n* (%)				
Yes	3 (1.1)	3 (1.3)	0 (0.0)	
No	274 (98.9)	235 (98.7)	39 (100.0)	0.999
Other surgical complication, *n* (%)				0.999
Yes	27 (9.8)	23 (9.7)	4 (10.3)	
No	250 (90.2)	215 (90.3)	35 (89.7)	

9,

**Table 10:** Univariable analysis; oesophagectomy all complications

Variable	Odds ratio	95% CI	*p* value
Age			0.032
< = 75 years	Reference		
> 75 years	2.21	1.07–4.57	
Sex			0.829
Female	Reference		
Male	1.07	0.59–1.92	
ASA Grade			0.013
1 or 2	Reference		
3 or 4	1.85	1.14–3.00	
COPD			0.045
No	Reference		
Yes	2.23	1.02–4.87	
HTN			0.871
No	Reference		
Yes	1.04	0.62–1.77	
CAD			0.016
No	Reference		
Yes	2.42	1.18–4.96	
Arrhythmia			0.045
No	Reference		
Yes	2.4	1.02–5.66	
Diabetes			0.581
No	Reference		
Yes	1.23	0.59–2.56	
PE/DVT			0.828
No	Reference		
Yes	0.88	0.28–2.80	
TIA/CVA			0.285
No	Reference		
Yes	2.12	0.54–8.36	
Smoking			0.775
No	Reference		
Yes	0.88	0.35–2.18	
Renal			0.17
No	Reference		
Yes	4.54	0.52–39.40	
Pre-surgery treatment			0.008
Chemo-radiotherapy	Reference		
Chemotherapy	0.45	0.26–0.77	
None	1	0.53–1.88	
Hospital Type			0.02
Private	Reference		
Public	1.91	1.11–3.29	
Procedure type			0.008
Ivor Lewis	Reference		
3 Stage	1.95	1.19–3.19	
T Stage			0.027
T0	Reference		
T1	0.98	0.40–2.40	
T2	0.74	0.28–1.98	
T3	0.4	0.17–0.95	
T4	0.48	0.13–1.81	
N Stage			0.096
N0	Reference		
N1	0.53	0.31–0.92	
N2	1.32	0.55–3.18	
N3	0.74	0.27–2.08	

^*ASA*^ American Society of Anaesthesiologists; *COPD* Chronic Obstruction Pulmonary Disease; *HTN* Hypertension; *CAD* Coronary artery disease; *PE* Pulmonary embolism; *DVT* Deep venous thrombosis; *TIA* Transient ischaemic attack; *CVA* Cerebrovascular accident

10,

**Table 11:** Univariable analysis; oesophagectomy non-surgical complications

Variable	Odds ratio	95% CI	*p* value
Age			0.001
< = 75 years	Reference		
> 75 years	1.23	1.59–6.38	
Sex			0.848
Female	Reference		
Male	1.06	0.57–1.97	
ASA grade			0.012
1 or 2	Reference		
3 or 4	1.9	1.15–3.14	
COPD			0.863
No	Reference		
Yes	1.07	0.50–2.28	
HTN			0.517
No	Reference		
Yes	0.83	0.48–1.45	
CAD			0.199
No	Reference		
Yes	1.56	0.79–3.05	
Arrhythmia			0.004
No	Reference		
Yes	3.26	1.46–7.29	
Diabetes			0.184
No	Reference		
Yes	1.65	0.79–3.43	
PE/DVT			0.273
No	Reference		
Yes	1.91	0.60–6.10	
TIA/CVA			0.106
No	Reference		
Yes	2.9	0.80–10.54	
Smoking			0.999
No	Reference		
Yes	1	0.38–2.59	
Renal			0.444
No	Reference		
Yes	1.88	0.37–9.51	
Pre-surgery treatment			0.033
Chemo-radiotherapy	Reference		
Chemotherapy	0.49	0.27–0.89	
None	1.07	0.57–2.01	
Hospital Type			0.009
Private	Reference		
Public	2.07	1.20–3.55	
Procedure type			0.009
Ivor Lewis	Reference		
3 Stage	2.04	1.19–3.49	
T Stage			0.01
T0	Reference		
T1	0.94	0.39–2.24	
T2	1	0.38–2.63	
T3	0.45	0.19–1.07	
T4	0.83	0.22–3.20	
N Stage			0.307
N0	Reference		
N1	0.76	0.42–1.37	
N2	1.46	0.62–3.43	
N3	1.85	0.66–5.21	

^*ASA*^ American Society of Anaesthesiologists; *COPD* Chronic Obstruction Pulmonary Disease; *HTN* Hypertension; *CAD* Coronary artery disease; *PE* Pulmonary embolism; *DVT* Deep venous thrombosis; *TIA* Transient ischaemic attack; *CVA* Cerebrovascular accident

11,

**Table 12:** Oesophageal cohort multivariable analysis for non-surgical complications

	Odds ratio	95% CI	*p* value
Age			< 0.001
< = 75 years	Reference		
> 75 years	3.70	1.79–7.64	
Hospital type			0.005
Private	Reference		
Public	2.24	1.27–3.94	
Procedure type			0.010
Ivor Lewis	Reference		
3 Stage	2.09	1.19–3.65	

^12^
